# 4-Oxo-2,4-diphenyl­butane­nitrile

**DOI:** 10.1107/S1600536812006137

**Published:** 2012-02-17

**Authors:** Alaa A.-M. Abdel-Aziz, Adel S. El-Azab, Seik Weng Ng, Edward R. T. Tiekink

**Affiliations:** a Department of Pharmaceutical Chemistry, College of Pharmacy, King Saud University, Riyadh 11451, Saudi Arabia; b Department of Medicinal Chemistry, Faculty of Pharmacy, University of Mansoura, Mansoura 35516, Egypt; c Department of Organic Chemistry, Faculty of Pharmacy, Al-Azhar University, Cairo 11884, Egypt; d Department of Chemistry, University of Malaya, 50603 Kuala Lumpur, Malaysia; e Chemistry Department, Faculty of Science, King Abdulaziz University, PO Box 80203 Jeddah, Saudi Arabia

## Abstract

The title mol­ecule, C_16_H_13_NO, is twisted, the dihedral angle between the terminal phenyl rings being 68.40 (6)°. In the crystal, C—H⋯O and C—H⋯N inter­actions lead to supra­molecular layers in the *bc* plane.

## Related literature

For background to the synthetic applications of 2,4-diaryl-4-oxo-butane­nitriles, see: Coudert *et al.* (1990 ▶, 1988 ▶); Iida *et al.* (2007 ▶). For the preparation of the title compound, see Coudert *et al.* (1990 ▶). For the structure of the meth­oxy derivative, see: Abdel-Aziz *et al.* (2012 ▶).
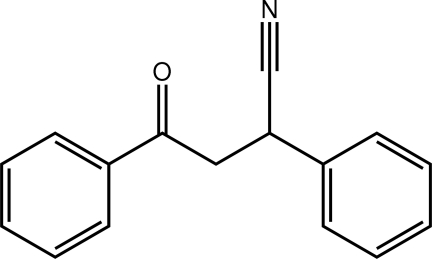


## Experimental

### 

#### Crystal data

C_16_H_13_NO
*M* *_r_* = 235.27
Monoclinic, 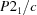

*a* = 14.2158 (3) Å
*b* = 8.9244 (2) Å
*c* = 9.7553 (2) Å
β = 99.217 (2)°
*V* = 1221.65 (5) Å^3^
*Z* = 4
Cu *K*α radiation
μ = 0.63 mm^−1^
*T* = 100 K
0.30 × 0.30 × 0.15 mm

#### Data collection

Agilent SuperNova Dual diffractometer with an Atlas detector
Absorption correction: multi-scan (*CrysAlis PRO*; Agilent, 2011 ▶) *T*_min_ = 0.752, *T*_max_ = 1.000
4625 measured reflections
2496 independent reflections
2187 reflections with *I* > 2σ(*I*)
*R*_int_ = 0.016

#### Refinement

*R*[*F* ^2^ > 2σ(*F* ^2^)] = 0.036
*wR*(*F* ^2^) = 0.097
*S* = 1.02
2496 reflections
163 parameters
H-atom parameters constrained
Δρ_max_ = 0.22 e Å^−3^
Δρ_min_ = −0.20 e Å^−3^

### 

Data collection: *CrysAlis PRO* (Agilent, 2011 ▶); cell refinement: *CrysAlis PRO*; data reduction: *CrysAlis PRO*; program(s) used to solve structure: *SHELXS97* (Sheldrick, 2008 ▶); program(s) used to refine structure: *SHELXL97* (Sheldrick, 2008 ▶); molecular graphics: *ORTEP-3* (Farrugia, 1997 ▶) and *DIAMOND* (Brandenburg, 2006 ▶); software used to prepare material for publication: *publCIF* (Westrip, 2010 ▶).

## Supplementary Material

Crystal structure: contains datablock(s) global, I. DOI: 10.1107/S1600536812006137/xu5469sup1.cif

Structure factors: contains datablock(s) I. DOI: 10.1107/S1600536812006137/xu5469Isup2.hkl

Supplementary material file. DOI: 10.1107/S1600536812006137/xu5469Isup3.cml

Additional supplementary materials:  crystallographic information; 3D view; checkCIF report

## Figures and Tables

**Table 1:** Hydrogen-bond geometry (Å, °)

*D*—H⋯*A*	*D*—H	H⋯*A*	*D*⋯*A*	*D*—H⋯*A*
C3—H3⋯N1^i^	0.95	2.62	3.3669 (17)	136
C8—H8b⋯O1^ii^	0.99	2.56	3.5246 (14)	163

Symmetry codes: (i) 
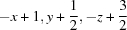
; (ii) 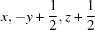.

**Fig. 1.**
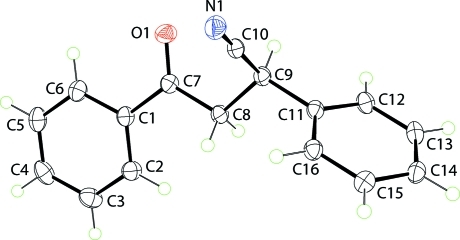
The molecular structure of (I) showing the atom-labelling scheme and displacement ellipsoids at the 50% probability level.

**Fig. 2.**
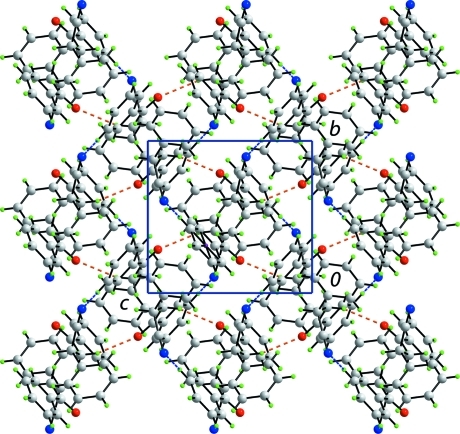
A view of the supramolecular in the bc plane in (I). The C—H···O and C—H···N interactions are shown as orange and blue dashed lines, respectively.

**Fig. 3.**
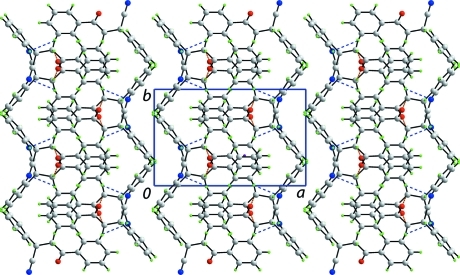
A view in projection down the c axis of the unit-cell contents for (I). The C—H···O and C—H···N interactions are shown as orange and blue dashed lines, respectively.

## supplementary crystallographic information

### Comment

2,4-Diaryl-4-oxo-butanenitriles constitute an important class of difunctional intermediates for both the synthesis of biologically active heterocycles, such as pyridazine derivatives, and as a source ketone (Coudert *et al.*, 1990; Coudert *et al.*, 1988; Iida *et al.*, 2007). Herein, the crystal structure of a 2,4-diaryl-4-oxo-butanenitrile derivative, 2,4-diphenyl-4-oxo-butanenitrile (I), is described. This compound has been prepared previously (Coudert *et al.*, 1990) and the structure of the methoxy derivative is known (Abdel-Aziz *et al.*, 2012).

The molecule of (I), Fig. 1, is twisted as seen in the value of the dihedral angle between the terminal benzene rings of 68.40 (6)°. The twist occurs between the C9—C11 bond [the C8—C9—C11—C12 torsion angle is 107.79 (12)°] with the other part of the molecule being relatively planar [the C7—C8—C9—C11 torsion angle is -179.69 (9)°].

Supramolecular layers in the *bc* plane are formed in the crystal packing *via* C—H···O and C—H···N interactions, Fig. 2 and Table 1. These stack along the *a* axis with no specific intermolecular interactions between the layers, Fig. 3.

### Experimental

Acetone cyanohydrin (0.045 mol) and 10% aqueous sodium carbonate (0.0015 mol and 1.5 ml water) were added to solution of benzalacetophenone (0.015 mol) in ethanol (50 ml). The mixture was heated at reflux temperature for 4 h. After cooling, the product which separated out was filtered off and recrystallized from methanol.

### Refinement

Carbon-bound H-atoms were placed in calculated positions [C—H = 0.95 to 1.00 Å, *U*_iso_(H) = 1.2*U*_eq_(C)] and were included in the refinement in the riding model approximation.

### Crystal data

**Table:**

C_16_H_13_NO	*F*(000) = 496
*M**_r_* = 235.27	*D*_x_ = 1.279 Mg m^−^^3^
Monoclinic, *P*2_1_/*c*	Cu *K*α radiation, λ = 1.5418 Å
Hall symbol: -P 2ybc	Cell parameters from 2233 reflections
*a* = 14.2158 (3) Å	θ = 3.2–76.0°
*b* = 8.9244 (2) Å	µ = 0.63 mm^−^^1^
*c* = 9.7553 (2) Å	*T* = 100 K
β = 99.217 (2)°	Prism, colourless
*V* = 1221.65 (5) Å^3^	0.30 × 0.30 × 0.15 mm
*Z* = 4	

### Data collection

**Table:**

Agilent SuperNova Dual diffractometer with an Atlas detector	2496 independent reflections
Radiation source: SuperNova (Cu) X-ray Source	2187 reflections with *I* > 2σ(*I*)
Mirror monochromator	*R*_int_ = 0.016
Detector resolution: 10.4041 pixels mm^-1^	θ_max_ = 76.2°, θ_min_ = 3.2°
ω scan	*h* = −17→17
Absorption correction: multi-scan (*CrysAlis PRO*; Agilent, 2011)	*k* = −11→6
*T*_min_ = 0.752, *T*_max_ = 1.000	*l* = −11→12
4625 measured reflections	

### Refinement

**Table:**

Refinement on *F*^2^	Primary atom site location: structure-invariant direct methods
Least-squares matrix: full	Secondary atom site location: difference Fourier map
*R*[*F*^2^ > 2σ(*F*^2^)] = 0.036	Hydrogen site location: inferred from neighbouring sites
*wR*(*F*^2^) = 0.097	H-atom parameters constrained
*S* = 1.02	*w* = 1/[σ^2^(*F*_o_^2^) + (0.048*P*)^2^ + 0.3439*P*] where *P* = (*F*_o_^2^ + 2*F*_c_^2^)/3
2496 reflections	(Δ/σ)_max_ < 0.001
163 parameters	Δρ_max_ = 0.22 e Å^−^^3^
0 restraints	Δρ_min_ = −0.20 e Å^−^^3^

### Special details

**Table:**

Geometry. All e.s.d.'s (except the e.s.d. in the dihedral angle between two l.s. planes) are estimated using the full covariance matrix. The cell e.s.d.'s are taken into account individually in the estimation of e.s.d.'s in distances, angles and torsion angles; correlations between e.s.d.'s in cell parameters are only used when they are defined by crystal symmetry. An approximate (isotropic) treatment of cell e.s.d.'s is used for estimating e.s.d.'s involving l.s. planes.
Refinement. Refinement of *F*^2^ against ALL reflections. The weighted *R*-factor *wR* and goodness of fit *S* are based on *F*^2^, conventional *R*-factors *R* are based on *F*, with *F* set to zero for negative *F*^2^. The threshold expression of *F*^2^ > σ(*F*^2^) is used only for calculating *R*-factors(gt) *etc*. and is not relevant to the choice of reflections for refinement. *R*-factors based on *F*^2^ are statistically about twice as large as those based on *F*, and *R*- factors based on ALL data will be even larger.

### Fractional atomic coordinates and isotropic or equivalent isotropic displacement parameters (Å^2^)

**Table:**

	*x*	*y*	*z*	*U*_iso_*/*U*_eq_	
O1	0.36576 (6)	0.21438 (10)	0.43914 (9)	0.0285 (2)	
N1	0.17432 (7)	0.10500 (12)	0.59790 (12)	0.0279 (2)	
C1	0.49902 (8)	0.30995 (13)	0.59134 (12)	0.0208 (2)	
C2	0.53560 (8)	0.42071 (14)	0.68608 (12)	0.0247 (3)	
H2	0.4947	0.4960	0.7124	0.030*	
C3	0.63208 (9)	0.42086 (16)	0.74205 (13)	0.0298 (3)	
H3	0.6570	0.4966	0.8062	0.036*	
C4	0.69199 (8)	0.31074 (16)	0.70442 (13)	0.0299 (3)	
H4	0.7575	0.3102	0.7441	0.036*	
C5	0.65640 (9)	0.20143 (15)	0.60905 (14)	0.0297 (3)	
H5	0.6976	0.1268	0.5826	0.036*	
C6	0.56030 (9)	0.20125 (14)	0.55228 (13)	0.0252 (3)	
H6	0.5361	0.1267	0.4865	0.030*	
C7	0.39562 (8)	0.30324 (13)	0.53106 (12)	0.0205 (2)	
C8	0.32829 (7)	0.40714 (13)	0.59087 (12)	0.0201 (2)	
H8	0.3436	0.5120	0.5698	0.024*	
H8B	0.3386	0.3957	0.6931	0.024*	
C9	0.22257 (7)	0.37701 (13)	0.53382 (12)	0.0198 (2)	
H9	0.2119	0.3901	0.4307	0.024*	
C10	0.19687 (8)	0.22196 (13)	0.56679 (12)	0.0211 (2)	
C11	0.15783 (7)	0.48444 (12)	0.59617 (12)	0.0187 (2)	
C12	0.11047 (8)	0.59901 (13)	0.51696 (12)	0.0218 (2)	
H12	0.1195	0.6126	0.4233	0.026*	
C13	0.04974 (8)	0.69400 (13)	0.57491 (13)	0.0232 (3)	
H13	0.0177	0.7726	0.5207	0.028*	
C14	0.03583 (8)	0.67459 (13)	0.71113 (13)	0.0224 (2)	
H14	−0.0067	0.7384	0.7496	0.027*	
C15	0.08427 (8)	0.56136 (13)	0.79140 (12)	0.0234 (2)	
H15	0.0755	0.5483	0.8852	0.028*	
C16	0.14544 (8)	0.46762 (12)	0.73418 (12)	0.0211 (2)	
H16	0.1792	0.3913	0.7895	0.025*	

### Atomic displacement parameters (Å^2^)

**Table:**

	*U*^11^	*U*^22^	*U*^33^	*U*^12^	*U*^13^	*U*^23^
O1	0.0216 (4)	0.0328 (5)	0.0314 (5)	−0.0005 (4)	0.0048 (3)	−0.0109 (4)
N1	0.0227 (5)	0.0233 (5)	0.0370 (6)	0.0003 (4)	0.0031 (4)	−0.0017 (4)
C1	0.0179 (5)	0.0224 (5)	0.0228 (5)	0.0004 (4)	0.0057 (4)	0.0020 (4)
C2	0.0196 (5)	0.0287 (6)	0.0265 (6)	−0.0012 (5)	0.0058 (4)	−0.0036 (5)
C3	0.0223 (6)	0.0390 (7)	0.0281 (6)	−0.0062 (5)	0.0038 (5)	−0.0029 (6)
C4	0.0165 (5)	0.0441 (8)	0.0290 (6)	−0.0001 (5)	0.0032 (5)	0.0087 (6)
C5	0.0217 (6)	0.0319 (7)	0.0365 (7)	0.0067 (5)	0.0081 (5)	0.0053 (5)
C6	0.0233 (6)	0.0239 (6)	0.0294 (6)	0.0020 (5)	0.0071 (5)	0.0007 (5)
C7	0.0187 (5)	0.0212 (5)	0.0223 (5)	−0.0007 (4)	0.0052 (4)	0.0003 (4)
C8	0.0155 (5)	0.0214 (5)	0.0233 (5)	−0.0004 (4)	0.0032 (4)	−0.0014 (4)
C9	0.0163 (5)	0.0210 (5)	0.0222 (5)	0.0011 (4)	0.0030 (4)	−0.0002 (4)
C10	0.0145 (5)	0.0231 (6)	0.0250 (5)	0.0026 (4)	0.0012 (4)	−0.0036 (5)
C11	0.0136 (5)	0.0177 (5)	0.0248 (5)	−0.0015 (4)	0.0029 (4)	−0.0007 (4)
C12	0.0171 (5)	0.0243 (6)	0.0241 (6)	−0.0001 (4)	0.0042 (4)	0.0043 (5)
C13	0.0167 (5)	0.0208 (5)	0.0317 (6)	0.0019 (4)	0.0023 (4)	0.0047 (5)
C14	0.0175 (5)	0.0193 (5)	0.0308 (6)	0.0011 (4)	0.0054 (4)	−0.0021 (5)
C15	0.0239 (6)	0.0220 (5)	0.0250 (6)	0.0006 (5)	0.0060 (4)	−0.0002 (5)
C16	0.0207 (5)	0.0180 (5)	0.0243 (6)	0.0007 (4)	0.0027 (4)	0.0012 (4)

### Geometric parameters (Å, º)

**Table:**

O1—C7	1.2212 (14)	C8—H8	0.9900
N1—C10	1.1470 (16)	C8—H8B	0.9900
C1—C2	1.3956 (17)	C9—C10	1.4796 (16)
C1—C6	1.3970 (16)	C9—C11	1.5217 (14)
C1—C7	1.4944 (15)	C9—H9	1.0000
C2—C3	1.3926 (17)	C11—C12	1.3889 (16)
C2—H2	0.9500	C11—C16	1.3936 (16)
C3—C4	1.3879 (19)	C12—C13	1.3929 (16)
C3—H3	0.9500	C12—H12	0.9500
C4—C5	1.386 (2)	C13—C14	1.3853 (17)
C4—H4	0.9500	C13—H13	0.9500
C5—C6	1.3894 (17)	C14—C15	1.3914 (16)
C5—H5	0.9500	C14—H14	0.9500
C6—H6	0.9500	C15—C16	1.3868 (16)
C7—C8	1.5151 (15)	C15—H15	0.9500
C8—C9	1.5403 (14)	C16—H16	0.9500
			
C2—C1—C6	119.34 (11)	H8—C8—H8B	107.7
C2—C1—C7	121.85 (10)	C10—C9—C11	108.43 (9)
C6—C1—C7	118.81 (11)	C10—C9—C8	110.20 (9)
C3—C2—C1	119.96 (11)	C11—C9—C8	111.29 (9)
C3—C2—H2	120.0	C10—C9—H9	109.0
C1—C2—H2	120.0	C11—C9—H9	109.0
C4—C3—C2	120.21 (12)	C8—C9—H9	109.0
C4—C3—H3	119.9	N1—C10—C9	176.20 (12)
C2—C3—H3	119.9	C12—C11—C16	119.46 (10)
C3—C4—C5	120.15 (11)	C12—C11—C9	120.78 (10)
C3—C4—H4	119.9	C16—C11—C9	119.76 (10)
C5—C4—H4	119.9	C11—C12—C13	119.93 (11)
C4—C5—C6	119.88 (12)	C11—C12—H12	120.0
C4—C5—H5	120.1	C13—C12—H12	120.0
C6—C5—H5	120.1	C14—C13—C12	120.41 (11)
C5—C6—C1	120.44 (12)	C14—C13—H13	119.8
C5—C6—H6	119.8	C12—C13—H13	119.8
C1—C6—H6	119.8	C13—C14—C15	119.79 (11)
O1—C7—C1	121.29 (10)	C13—C14—H14	120.1
O1—C7—C8	120.89 (10)	C15—C14—H14	120.1
C1—C7—C8	117.79 (10)	C16—C15—C14	119.82 (11)
C7—C8—C9	113.21 (9)	C16—C15—H15	120.1
C7—C8—H8	108.9	C14—C15—H15	120.1
C9—C8—H8	108.9	C15—C16—C11	120.55 (10)
C7—C8—H8B	108.9	C15—C16—H16	119.7
C9—C8—H8B	108.9	C11—C16—H16	119.7
			
C6—C1—C2—C3	−0.85 (18)	C7—C8—C9—C10	59.99 (12)
C7—C1—C2—C3	178.58 (11)	C7—C8—C9—C11	−179.69 (9)
C1—C2—C3—C4	−0.31 (19)	C10—C9—C11—C12	−130.84 (11)
C2—C3—C4—C5	1.11 (19)	C8—C9—C11—C12	107.79 (12)
C3—C4—C5—C6	−0.74 (19)	C10—C9—C11—C16	48.89 (13)
C4—C5—C6—C1	−0.44 (19)	C8—C9—C11—C16	−72.47 (13)
C2—C1—C6—C5	1.23 (18)	C16—C11—C12—C13	−1.27 (16)
C7—C1—C6—C5	−178.23 (11)	C9—C11—C12—C13	178.47 (10)
C2—C1—C7—O1	173.35 (11)	C11—C12—C13—C14	−0.31 (17)
C6—C1—C7—O1	−7.21 (17)	C12—C13—C14—C15	1.28 (17)
C2—C1—C7—C8	−8.53 (16)	C13—C14—C15—C16	−0.67 (17)
C6—C1—C7—C8	170.91 (10)	C14—C15—C16—C11	−0.91 (17)
O1—C7—C8—C9	5.29 (15)	C12—C11—C16—C15	1.88 (17)
C1—C7—C8—C9	−172.83 (9)	C9—C11—C16—C15	−177.85 (10)

### Hydrogen-bond geometry (Å, º)

**Table:**

*D*—H···*A*	*D*—H	H···*A*	*D*···*A*	*D*—H···*A*
C3—H3···N1^i^	0.95	2.62	3.3669 (17)	136
C8—H8b···O1^ii^	0.99	2.56	3.5246 (14)	163

Symmetry codes: (i) −*x*+1, *y*+1/2, −*z*+3/2; (ii) *x*, −*y*+1/2, *z*+1/2.
